# Supplementary material for: Mechanisms governing the pioneering and redistribution capabilities of the non-classical pioneer PU.1
Source: Nat Commun. 2020 Jan 21;11:402. doi: 10.1038/s41467-019-13960-2 (PMC6972792; doi:10.1038/s41467-019-13960-2)
Supplement: Supplementary file 7 — Source data [file 41467_2019_13960_MOESM7_ESM.zip › Source_Data/Figure6/Figure6B_MotifScanOutput/ETS1_mutPU.1/homerResults/motif27.similar.html]

motif27

## Information for motif27

G
A
C
T
C
G
A
T
G
T
A
C
A
G
T
C
A
G
T
C
T
C
G
A
A
T
C
G
G
T
C
A
C
T
G
A
  
Reverse Opposite:  

G
A
C
T
A
C
G
T
T
A
G
C
A
G
C
T
C
T
A
G
C
T
A
G
A
C
T
G
G
C
T
A
C
T
G
A
  

|  |  |
| --- | --- |
| p-value: | 1e-83 |
| log p-value: | -1.932e+02 |
| Information Content per bp: | 1.709 |
| Number of Target Sequences with motif | 1031.0 |
| Percentage of Target Sequences with motif | 7.65% |
| Number of Background Sequences with motif | 1171.6 |
| Percentage of Background Sequences with motif | 3.34% |
| Average Position of motif in Targets | 100.2 +/- 52.7bp |
| Average Position of motif in Background | 100.4 +/- 63.7bp |
| Strand Bias (log2 ratio + to - strand density) | 0.2 |
| Multiplicity (# of sites on avg that occur together) | 1.05 |
| Motif File: | file (matrix) reverse opposite |

### Similar de novo motifs found

|  |  |  |  |  |  |  |  |
| --- | --- | --- | --- | --- | --- | --- | --- |
| Rank | Match Score | Redundant Motif | P-value | log P-value | % of Targets | % of Background | Motif file |
| 1 | 0.922 | T C G A G A C T A C G T A C G T A G T C G A C T T A G C C T G A A C T G C G T A C G T A | 1e-83 | -191.764844 | 6.38% | 2.53% | motif file (matrix) |
| 2 | 0.929 | A T C G C T G A A G C T A C G T A C G T A G T C A G C T C T G A C T G A A C T G C G T A C G T A T C G A | 1e-82 | -189.458386 | 6.04% | 2.33% | motif file (matrix) |
| 3 | 0.892 | A C G T A C G T A G T C G A C T T A C G C T G A A C T G C G T A C G T A C T G A G A C T T A G C | 1e-80 | -185.771952 | 6.44% | 2.61% | motif file (matrix) |
| 4 | 0.917 | G A T C A C G T C G A T T A G C G A C T T C G A C T G A A C T G G C T A C G T A | 1e-78 | -181.301532 | 6.89% | 2.94% | motif file (matrix) |
| 5 | 0.848 | C G A T C A T G C G T A G A C T A C G T A C G T A G T C A C G T T A C G C G T A A C T G C G T A C G T A C T G A | 1e-54 | -125.354905 | 3.39% | 1.17% | motif file (matrix) |
| 6 | 0.832 | A C G T A G T C G C A T A G T C C G T A A T C G C G T A C T G A | 1e-40 | -93.969338 | 15.14% | 10.63% | motif file (matrix) |
